# Rationale and design of the German-Speaking Myeloma Multicenter Group (GMMG) trial ReLApsE: a randomized, open, multicenter phase III trial of lenalidomide/dexamethasone versus lenalidomide/dexamethasone plus subsequent autologous stem cell transplantation and lenalidomide maintenance in patients with relapsed multiple myeloma

**DOI:** 10.1186/s12885-016-2321-2

**Published:** 2016-04-25

**Authors:** Marc-Andrea Baertsch, Jana Schlenzka, Elias K. Mai, Maximilian Merz, Jens Hillengaß, Marc S. Raab, Dirk Hose, Patrick Wuchter, Anthony D. Ho, Anna Jauch, Thomas Hielscher, Christina Kunz, Steffen Luntz, Stefan Klein, Ingo G. H. Schmidt-Wolf, Martin Goerner, Martin Schmidt-Hieber, Peter Reimer, Ullrich Graeven, Roland Fenk, Hans Salwender, Christof Scheid, Axel Nogai, Mathias Haenel, Hans W. Lindemann, Hans Martin, Richard Noppeney, Katja Weisel, Hartmut Goldschmidt

**Affiliations:** Hematology, Oncology and Rheumatology, University Hospital Heidelberg, Heidelberg, Germany; Institute for Human Genetics, University of Heidelberg, Heidelberg, Germany; Division of Biostatistics, German Cancer Research Center, Heidelberg, Germany; Coordination Centre for Clinical Trials (KKS), University Hospital Heidelberg, Heidelberg, Germany; Hematology and Oncology, University Hospital Mannheim, Mannheim, Germany; Center for Integrated Oncology, Med. Klinik und Poliklinik III, University Hospital Bonn, Bonn, Germany; Hematology, Oncology and Palliative Care, Community Hospital Bielefeld, Bielefeld, Germany; Hematology and Oncology, Helios-Hospital Berlin Buch, Berlin, Germany; Hematology, Oncology and Stem Cell Transplantation, Evangelisches Krankenhaus Essen-Werden gGmbH, Essen, Germany; Hematology, Oncology and Gastroenterology, Maria-Hilf-Krankenhaus, Mönchengladbach, Germany; Hematology, Oncology and Clinical Immunology, University of Duesseldorf, Duesseldorf, Germany; Hematology, Oncology and Palliative Care, Asklepios Klinik Altona, Hamburg, Germany; Department I of Internal Medicine, University of Cologne, Cologne, Germany; Internal Medicine III, Charité Campus Benjamin Franklin, Berlin, Germany; Hematology, Oncology and Stem Cell Transplantation, Klinikum Chemnitz GmbH, Chemnitz, Germany; Hematology and Oncology, Kath. Krankenhaus Hagen gem. GmbH - St.-Marien-Hospital, Hagen, Germany; Hematology and Oncology, Goethe University, Frankfurt, Germany; Hematology, University Hospital Essen, Essen, Germany; Hematology, Oncology and Immunology, University of Tuebingen, Tuebingen, Germany; National Center for Tumor Diseases, Heidelberg University Hospital, Heidelberg, Germany

**Keywords:** Multiple myeloma, Relapse, Second-line treatment, Lenalidomide, Autologous stem cell transplantation, High-dose chemotherapy

## Abstract

**Background:**

Despite novel therapeutic agents, most multiple myeloma (MM) patients eventually relapse. Two large phase III trials have shown significantly improved response rates (RR) of lenalidomide/dexamethasone compared with placebo/dexamethasone in relapsed MM (RMM) patients. These results have led to the approval of lenalidomide for RMM patients and lenalidomide/dexamethasone has since become a widely accepted second-line treatment. Furthermore, in RMM patients consolidation with high-dose chemotherapy plus autologous stem cell transplantation has been shown to significantly increase progression free survival (PFS) as compared to cyclophosphamide in a phase III trial. The randomized prospective ReLApsE trial is designed to evaluate PFS after lenalidomide/dexamethasone induction, high-dose chemotherapy consolidation plus autologous stem cell transplantation and lenalidomide maintenance compared with the well-established lenalidomide/dexamethasone regimen in RMM patients.

**Methods/Design:**

ReLApsE is a randomized, open, multicenter phase III trial in a planned study population of 282 RMM patients. All patients receive three lenalidomide/dexamethasone cycles and - in absence of available stem cells from earlier harvesting - undergo peripheral blood stem cell mobilization and harvesting. Subsequently, patients in arm A continue on consecutive lenalidomide/dexamethasone cycles, patients in arm B undergo high dose chemotherapy plus autologous stem cell transplantation followed by lenalidomide maintenance until discontinuation criteria are met. Therapeutic response is evaluated after the 3^rd^ (arm A + B) and the 5^th^ lenalidomide/dexamethasone cycle (arm A) or 2 months after autologous stem cell transplantation (arm B) and every 3 months thereafter (arm A + B). After finishing the study treatment, patients are followed up for survival and subsequent myeloma therapies. The expected trial duration is 6.25 years from first patient in to last patient out. The primary endpoint is PFS, secondary endpoints include overall survival (OS), RR, time to best response and the influence of early versus late salvage high dose chemotherapy plus autologous stem cell transplantation on OS.

**Discussion:**

This phase III trial is designed to evaluate whether high dose chemotherapy plus autologous stem cell transplantation and lenalidomide maintenance after lenalidomide/dexamethasone induction improves PFS compared with the well-established continued lenalidomide/dexamethasone regimen in RMM patients. Trial registration: ISRCTN16345835 (date of registration 2010-08-24).

**Electronic supplementary material:**

The online version of this article (doi:10.1186/s12885-016-2321-2) contains supplementary material, which is available to authorized users.

## Background

With an incidence rate of 5-6/100.000, multiple myeloma (MM) accounts for 13 % of hematologic cancers and 1 % of all neoplastic diseases in Western countries [1]. In first-line treatment of MM, high-dose chemotherapy (HDCT) with subsequent autologous stem-cell transplantation (ASCT) has become the standard of care in eligible patients [1–3]. In recent years patient outcomes have been further improved by the introduction of novel agents, namely immunomodulatory drugs (IMiDs) and proteasome inhibitors (PIs) into first-line therapy. Nevertheless, MM remains largely incurable and almost all patients eventually relapse. Therapeutic options in the setting of relapsed MM (RMM) include salvage HDCT/ASCT, novel agents (IMiDs, PIs, agents under clinical investigation), chemotherapy, corticosteroids, and allogeneic stem cell transplantation [4]. In the absence of an established standard of care the selection of a suitable treatment regimen for RMM patients is usually based on disease- and patient-specific factors such as regimens employed in and response to prior lines of therapy. Again, the introduction of IMIDs and proteasome inhibitors into the treatment of RMM has led to a major increase in survival rates of this patient population [4–11].

Lenalidomide is an orally administered IMiD and a derivative of the structurally related thalidomide [12]. The mechanism of action of lenalidomide is multifaceted [13, 14] including apoptosis induction, alteration of the interaction of myeloma cells with bone marrow stroma [15], antiangiogenesis [16, 17], and immunomodulation [18, 19]. More recently, anti-myeloma activity of lenalidomide has been shown to depend on interaction with cereblon, a protein involved in the teratogenicity of thalidomide [20–22]. Two pivotal phase III trials (MM-009 and MM-010) have demonstrated highly significant improvement of response rates with lenalidomide/dexamethasone compared with placebo/dexamethasone in a total of 704 RMM patients and subsequently led to regulatory approval of lenalidomide in the USA and the EU. All patients in these pivotal trials received 40 mg dexamethasone on days 1–4, 9–12 and 17–20 and either 25 mg lenalidomide or placebo on days 1–21 of 28-day cycles. After completion of the 4^th^ cycle, dexamethasone was limited to days 1–4 [8, 9]. Pooled analysis of both registration studies revealed superior overall response rate (ORR; 60.6 % vs. 21.9 %), time to progression (TTP; 13.4 vs. 4.6 months) and median OS (38.0 vs. 31.6 months) of the lenalidomide-containing regimen, but also significantly more adverse events (AEs), especially grade 3–4 neutropenia (35.4 % vs. 3.4 %) and thromboembolic events (15.9 % vs. 5.4 %) [23]. Dexamethasone dosing was subject of investigation in a phase III trial in NDMM patients. In combination with lenalidomide, high-dose dexamethasone (480 mg/cycle; 40 mg on days 1–4, 9–12 and 17–20) led to significantly increased ORR (79 % vs. 68 %), but low dose-dexamethasone (160 mg/cycle; 40 mg on days 1, 8, 15, 22) showed significantly increased OS after 1 and 2 years (96 % vs. 87 % and 87 % vs. 75 %, respectively). This was related to a significantly lower incidence of AEs including infectious and thromboembolic events and deaths in the low-dose dexamethasone arm [24]. A subgroup analysis of RMM patients from MM-009 and MM-010 with dexamethasone dose reduction confirmed these results [25]. Furthermore, lenalidomide maintenance treatment has been evaluated in four phase III trials in NDMM patients [26–29]. While PFS after lenalidomide maintenance was markedly prolonged in all four trials, OS was significantly superior in only one trial [30]. Despite the finding of an increased incidence of second primary malignancies in the maintenance arm, the odds for lower mortality are in favor of lenalidomide maintenance treatment [31].

Available data on HDCT/ASCT in RMM patients largely comes from retrospective, non-randomized, single-center or registry-based studies [32–45] and few prospective trials of early (i.e. first-line) vs. delayed (i.e. second-line) HDCT/ASCT [46, 47]. These studies suggest that salvage HDCT/ASCT is feasible in RMM patients considered eligible based on sufficient general condition and absence of prohibitive comorbidities. However, reported outcomes vary due to small and/or heterogeneous patient populations and differing salvage regimens. A recently published review of 19 retrospective studies of salvage HDCT/ASCT in patients treated with first-line HDCT/ASCT reported median ORR of 64.3 % (range 27.3 to 97.4 %), median PFS of 12.3 months (range 6 to 36 months), median OS of 32.4 months (range 8 to 79.1 months) and median transplantation associated mortality (TRM) of 4.1 % (range 0 to 22 %). TTP after first-line HDCT/ASCT of ≥ 19.8 months (range 6 to 36 months) was identified as the major predictive factor for a beneficial outcome after salvage HDCT/ASCT. Furthermore, the quality of response after first-line HDCT/ASCT and the number of prior lines of therapy were suggested as predictive factors [48]. A similar, retrospective study from our center involving 200 patients treated between 1995 and 2010 with salvage HDCT/ASCT at relapse after first-line HDCT/ASCT yielded comparable results with median ORR of 80.4 %, median PFS of 15.4 months, median OS of 42.3 months and TRM of 3 %. An overall survival advantage for patients treated with bortezomib or lenalidomide during reinduction suggested that salvage HDCT/ASCT and novel agents are complementary treatment approaches. Moreover, a favorable cytogenetic status (i.e. absence of +1q21, del(17p13) and t(4;14)) was associated with significantly prolonged PFS (25.6 vs. 13.2 months) [41]. Recently, results from the first prospective, randomized trial on salvage HDCT/ASCT in patients with a previous HDCT/ASCT have been published [49]. At first relapse at least 18 months (later reduced to 12 months) after previous HDCT/ASCT, 293 patients were treated with bortezomib/doxorubicin/dexamethasone (PAD) reinduction therapy and underwent stem cell mobilization and harvesting if applicable. Subsequently, 174 eligible patients were randomized on a 1:1 basis to receive either HDCT (melphalan 200 mg/m^2^) and ASCT or cyclophosphamide (400 mg/m^2^ per week for 12 weeks) consolidation therapy. ORR in the HDCT/ASCT arm was 83 % compared with 75 % in the cyclophosphamide arm with significantly more very good partial remissions or better after HDCT/ASCT (60 % vs. 47 %). The primary endpoint of median TTP was significantly prolonged (19 vs. 11 months). OS did not differ significantly with median OS not having been reached at the cut off date for the final analysis and 3-year OS of 80.3 % vs. 62.9 %. TRM was 1 % in the HDCT/ASCT arm. A subgroup with an unfavorable cytogenetic status (i.e. t(4;14), t(14;16) and/or del(17p13)) did not benefit from HDCT/ASCT (hazard ratio (HR) 2.41). However, the low proportion of patients with available cytogenetic data (51 % of patients undergoing randomization) and the low number of patients with an unfavorable cytogenetic status (*n* = 13; 15 %) limit the interpretation of this finding.

Existing evidence suggests both feasibility and benefit of salvage HDCT/ASCT in eligible RMM patients. Moreover, novel agent based regimens and HDCT/ASCT seem to be complementary salvage treatment approaches. The ReLApsE trial is designed to analyze the benefit of salvage HDCT/ASCT incorporated into the widely used and novel agent based salvage treatment lenalidomide/dexamethasone (Rd) in a prospective, randomized setting.

## Methods/Design

### Design

ReLApsE is a randomized, controlled, open-label, multicenter phase III trial in a planned study population of 282 RMM patients in their 1^st^ to 3^rd^ relapse. Patients are randomized 1:1 to receive either Rd reinduction, HDCT/ASCT and lenalidomide maintenance or continued Rd. Patients are stratified according to study site and HDCT/ASCT during first-line therapy (yes vs. no). The protocol published here is based on the full protocol version 4.0 as of 2014-04-15.

### Trial objectives

#### Primary objective

PFS is defined as time from randomization to progressive disease (PD) or death, irrespective of the cause of death, and is evaluated as primary objective. Patients that are event-free at the time of analysis are censored at the date of the last response evaluation.

#### Secondary objectives

Secondary objectives are: OS; response rates to Rd, HDCT/ASCT and lenalidomide maintenance; time to best response; impact of complete response (CR) and very good partial response (VGPR) prior to HDCT/ASCT and prior to lenalidomide maintenance on PFS and OS; impact of early salvage HDCT/ASCT with subsequent lenalidomide maintenance versus late salvage HDCT/ASCT (performed as a post-study treatment) on OS; feasibility of stem cell mobilization and apheresis; safety and toxicity (type, frequency, CTC grading, causality of AEs); time to initiation of next anti-myeloma treatment.

### Setting

ReLApsE is an investigator initiated trial designed and carried out by the German-Speaking Myeloma Multicenter Group (GMMG). The trial setting is multicentric with 16 participating study sites located in Germany: Helios Hospital Berlin-Buch; Charité Campus Benjamin Franklin, Berlin; Community Hospital Bielefeld; University Hospital Bonn; Klinikum Chemnitz GmbH; University Hospital Düsseldorf; University Hospital Essen; Evangelisches Krankenhaus Essen-Werden gGmbH; Goethe University, Frankfurt; Katholisches Krankenhaus Hagen gGmbH; Asklepios Klinik Altona, Hamburg; Heidelberg University Hospital (trial sponsor); University Hospital Cologne; University Hospital Mannheim; Maria-Hilf-Krankenhaus Mönchengladbach; University Hospital Tübingen.

### Estimated timeline

Recruitment of study patients was initiated during the 4^th^ quarter of 2010 (first patient in; FPI) and is planned to be completed during the 4^th^ quarter of 2015 (last patient in; LPI). The trial is planned to be finished during the 1^st^ quarter of 2017 (last patient out; LPO), 1.25 years after LPI. An interim analysis is planned after 96 PFS relevant events. The final report is scheduled for the 1^st^ quarter of 2018. Patients with continued benefit from treatment at the end of the trial will be allowed to continue treatment. For patients in arm B receiving maintenance lenalidomide at the end of the trial, Celgene provides lenalidomide until disease progression at no cost.

### Ethical aspects, informed consent, and safety

All study procedures are in accordance with International Conference on Harmonization of good clinical practice (ICH-GCP) guidelines, the declaration of Helsinki, and German laws, regulations and organizations. Documented approval from the ethics committees/institutional review boards (IRB) of the Medical Faculty of the Heidelberg University (main IRB) and all participating study sites has been obtained prior to study start and a data safety monitoring board (DSMB) has been installed to monitor the trial.

Written informed consent from each patient is obtained before any study-specific procedures are performed. Study participation and date of informed consent are documented in each patient’s files.

AEs are recorded in the patient’s case report form and relatedness to the study medication, intensity (according to CTCAE v4.03) and severity are classified. Serious AEs (SAEs) are recorded on an additional SAE form and are reported to the responsible safety officer within 24 h of detection. Suspected unexpected serious adverse reactions (SUSARs) are reported to the responsible ethics committee, the federal authorities and all investigators.

Due to the teratogenicity of lenalidomide a pregnancy prevention program was implemented.

### Selection of trial patients

Inclusion and exclusion criteria are listed in Table 1.

**Table 1:** Inclusion and exclusion criteria

*Inclusion criteria*
• Understanding of the nature and consequences of the trial and voluntary signature of the informed consent document
• Age ≥ 18 and ≤ 75 years at the time of consent and randomization
• Availability of stem cells from earlier harvesting if age ≥ 71 years
• 1^st^ to 3^rd^ relapse of symptomatic MM (according to IMWG criteria [51])
• Salmon and Durie stage [58] II or III
• Duration of response ≥ 12 months in case of first-line HDCT/ASCT
• WHO performance status (WHO PS) ≤ 2
• Laboratory findings within the following ranges ○ Absolute neutrophil count ≥ 1/nl ○ Platelet count ≥ 75/nl (depending on bone marrow infiltration with myeloma cells, platelet count ≥ 30/nl may be acceptable) ○ Creatinine clearance ≥ 30 ml/min ○ Total bilirubin ≤ 2 x the upper limit of normal (ULN; except for elevations caused by MM) ○ Alanine aminotransferase (ALT) ≤ 3 x ULN (except for elevations caused by MM)
• Absence of malignant diseases other than MM for ≥ 5 years (except basal-cell carcinoma and carcinoma in situ of the skin, the cervix and the breast)
• Ability to apply thrombosis prophylaxis
• Consent to all protocol requirements, especially those regarding the trial visit schedule and the pregnancy prevention program
*Exclusion criteria*
• Pregnant or breastfeeding female
• Previous treatment with lenalidomide, if: ○ Refractory (i.e. stable disease (SD) or progressive disease (PD) on treatment or ≤ 60 days after the end of treatment) ○ PD ≤ 6 months after the end of treatment if patient had responded (i.e. ≥ MR)
• Previous salvage HDCT/ASCT
• Known hypersensitivity to thalidomide, lenalidomide or components of lenalidomide
• Erythema nodosum as an exfoliative rash while on thalidomide
• Exposure to any other experimental substance within 28 days prior to enrollment
• Non-secretory MM (with normal free light chain ratio) that cannot be monitored by radiographic (e.g. MRI) examination
• Systemic amyloidosis with organ involvement (with the exception of AL-amyloidosis of the skin and/or bone marrow)
• Plasma cell leukemia
• Previous allogeneic stem cell transplantation
• Active, uncontrolled infectious disease
• Known positivity for HIV, hepatitis B or C
• Congestive heart failure (NYHA ≥ 3)
• Severe pulmonary, neurologic or psychiatric disease

### Trial procedures

An overview of the trial procedures and a checklist according to “Standard Protocol Items: Recommendations for Interventional Trials” (SPIRIT) guidelines are provided as Additional files 1 and 2.

#### Screening

The following diagnostic investigations are performed at screening to determine patient eligibility for study participation and to assess disease status before study treatment: patient history and physical examination (including body weight, height, WHO performance score (PS), and concomitant diseases), laboratory investigations (complete blood count including absolute neutrophil count (ANC), electrolytes, renal parameters, hepatic parameters, thyroid stimulating hormone, C reactive protein, lactate dehydrogenase, albumin, total protein, pregnancy test if applicable, β-2 microglobulin, immunoglobulins, monoclonal protein and free light chains in serum, monoclonal protein in urine, immunofixation in serum and urine), bone marrow aspiration (cytology, interphase fluorescence in situ hybridization (iFISH) in CD138-purified plasma cells as described previously [50]) radiographic imaging of the skeleton (low dose, whole body computed tomography (CT) or conventional X-ray imaging; appropriate imaging for disease quantification in case of non-secretory myeloma), electrocardiogram (ECG) and echocardiogram (exercise ECG if clinically indicated).

#### Study visits

Study visits are scheduled at the respective study site after the initial 3 Rd cycles, after Rd cycle 5 (arm A) or 2 months after HDCT/ASCT (arm B), every three months thereafter and at the end of study participation. Diagnostic investigations performed at these visits for assessment of efficacy and safety, as well as patient eligibility to continue study treatment include: patient history and physical examination (including AEs, WHO PS, signs of thrombosis, assessment of soft tissue plasmacytomas), laboratory investigations (listed in *Screening*), bone marrow aspiration (only if CR or PD are suspected), radiographic imaging of the skeleton (if clinically indicated or at least once a year; more frequently for response assessment in non-secretory MM), and ECG and echocardiography (after the initial 3 Rd cycles, before HDCT and if clinically indicated). Additionally, complete blood counts including ANC are determined weekly during Rd cycles 1 and 2 and every 2 to 4 weeks thereafter for safety reasons. During HDCT/ASCT safety investigations are performed according to study site standards.

#### Trial treatment

**Fig. 1:**
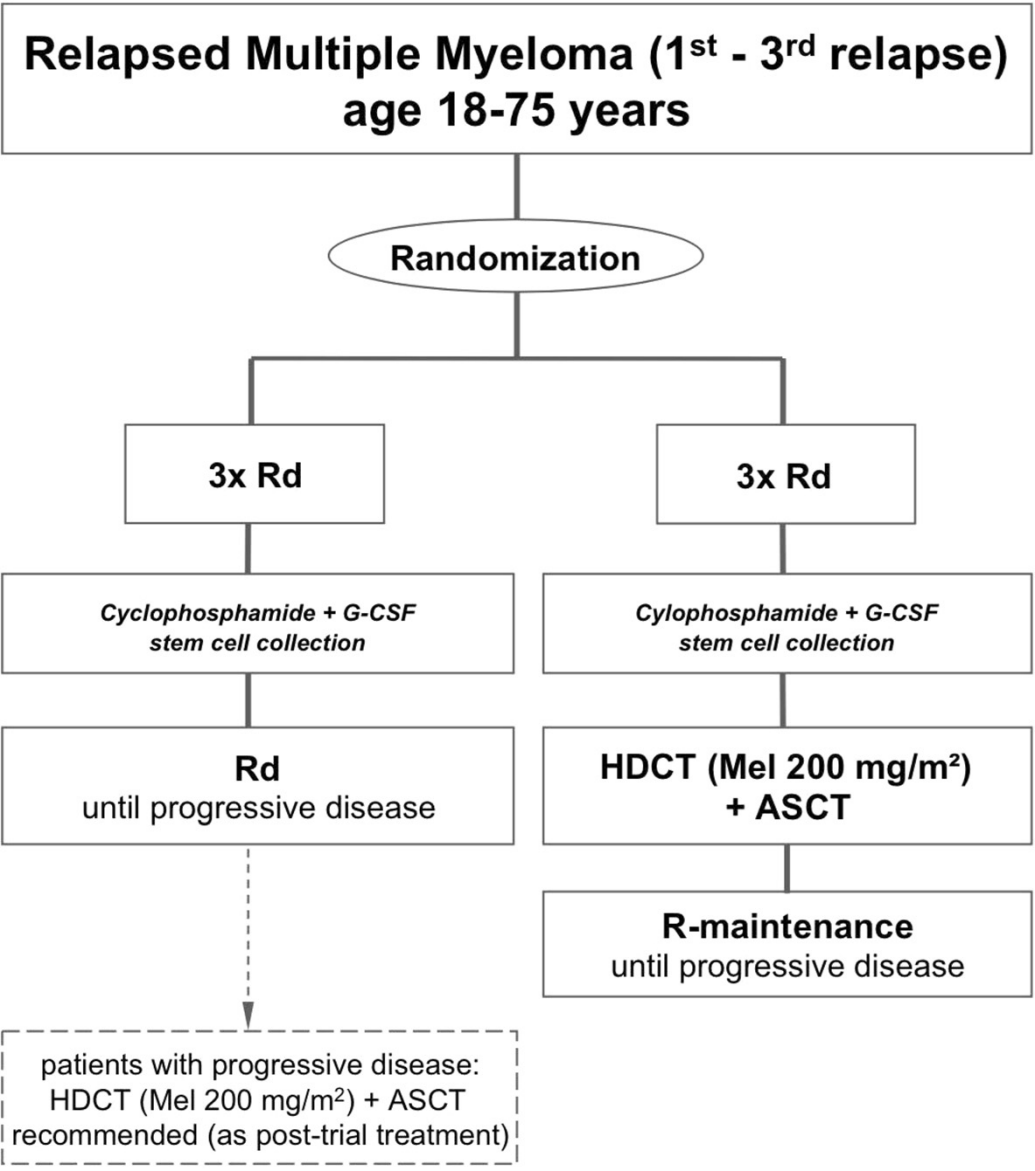
ReLApsE trial overview. *Rd*: 28 day cycle of lenalidomide (25 mg p.o. daily on days 1-21) and low-dose dexamethoasone (40 mg p.o. daily on days 1, 8, 15, 22); *cyclophosphamide*: 2 g*m^−2^ i.v. daily on days 1 and 2; *G-CSF*: Filgrastim 10 μg*kg^−1^*d^−1^ or lenograstim 300 μg*m^−2^*d^−1^ s.c. daily from day 5 until the end of apheresis; *HDCT*: High dose chemotherapy (melphalan 100 mg*m^−2^ i.v. daily on days -3 and -2); *ASCT*: Autologous stem cell transplantation (≥ 2*10^6^ CD34+ cells*kg bw^−1^ on day 0); *R-maintenance*: Lenalidomide maintenance (10 mg p.o. daily)

An overview of the treatment schedule is provided in Fig. 1. Following randomization, all patients receive reinduction treatment consisting of 3 Rd cycles of 28 days each (oral lenalidomide 25 mg on days 1–21, oral dexamethasone 40 mg on days 1, 8, 15, 22). Subsequently, all patients that do not have available stem cells from earlier harvesting (≥2*10^6^ CD34+ cells*kg bw^−1^) undergo peripheral blood stem cell mobilization and harvesting. Stem cell mobilization consists of cyclophosphamide (2 g*m^−2^ i.v. daily on days 1 and 2) and G-CSF (filgrastim 10 μg*kg^−1^*d^−1^ or lenograstim 300 μg*m^−2^*d^−1^ s.c. from day 5 until the end of apheresis); if unsuccessful, rescue mobilization with plerixafor is recommended. Determination of CD34+ cells in peripheral blood and leukapheresis are performed according to study site standard.

Patients in arm A then continue on consecutive Rd cycles (same dosages and intervals as reinduction treatment) until termination criteria are met.

Patients in arm B are examined for HDCT/ASCT eligibility (available stem cells; WHO PS ≤ 2; absence of severe pulmonary, neurologic, or psychiatric disease; transaminases and bilirubin ≤ 2,5 ULN; NYHA ≤ 2) and - if eligible - undergo HDCT (melphalan 100 mg*m^−2^ i.v. daily on days -3 and -2) and autologous stem cell transplantation (≥ 2*10^6^ CD34+ cells*kg bw^−1^ i.v. on day 0) no later than 5 weeks after the end of reinduction treatment or stem cell harvesting. In the absence of ANC < 1/nl, platelets < 30/nl, active infections and PD, maintenance treatment with oral lenalidomide (10 mg daily) is initiated no later than 8 weeks after ASCT. Maintenance treatment is continued until termination criteria are met.

In the case of toxicities (e.g. grade 3/4 neutropenia, thrombocytopenia, non-hematologic toxicities) attributed to the study medication, doses of lenalidomide and/or dexamethasone are reduced according to predefined algorithms based on the prescribing information. All medication is adapted to renal function according to the prescribing information if necessary. Melphalan is reduced to a single dose of 100 mg*m^−2^ (day -3) if creatinine clearance is ≤ 40 ml*min^−1^.

#### Supportive treatment

During cycle 1 of the reinduction treatment, all patients receive antibiotic prophylaxis with ciprofloxacin (500 mg twice daily). Antibiotic prophylaxis after cycle 1 is optional and based on clinical judgment. During reinduction treatment, all patients with platelets ≥ 50/nl receive thrombosis prophylaxis with low molecular weight heparin (LMWH; enoxaparin 40 mg s.c. daily). In arm A, thrombosis prophylaxis is switched to oral acetylsalicylic acid (100 mg daily) after Rd cycle 3 if no risk factors for thrombotic events are present; otherwise prophylaxis with LMWH is continued throughout the study treatment. During maintenance treatment in arm B thrombosis prophylaxis is not mandatory; however, it may be instituted based on clinical judgment.

Supportive treatment during stem cell mobilization/harvesting and HDCT/ASCT is performed according to study site standards.

Moreover, all patients are recommended to receive bisphosphonates (zoledronic acid 4 mg every 4 weeks i.v.). If VGPR is reached, duration of bisphosphonates should be limited to a total of 2 years. Due to the risk of bisphosphonate related osteonecrosis of the jaw, regular dental prophylaxis and temporary discontinuation of bisphosphonates as well as antibiotic prophylaxis in the context of invasive dental procedures are recommended.

#### Concomitant medication and treatment

Antibiotic prophylaxis, thrombosis prophylaxis and bisphosphonate treatment are administered as described above. Furthermore, red blood cell and platelet transfusions, G-CSF administration, immunoglobulin substitution, treatment of myeloma- or treatment-associated complications, radiotherapy for the treatment of myeloma-induced pain, and glucocorticoids up to 10 mg of prednisone for the treatment of concomitant diseases are permitted. Substances with antineoplastic activity other than those administered according to the trial protocol are not allowed.

#### Follow up

All patients are followed up after the end of study treatment on a regular basis, irrespective of the reason for discontinuation. During follow up, data on survival, toxicities, efficacy and subsequent myeloma-specific treatment including salvage HDCT/ASCT are collected.

### Response assessment

Disease response is determined based on International Myeloma Working Group (IMWG) criteria [51]. Minimal response (MR) as defined in the European Group for Bone and Marrow Transplantation (EBMT) criteria [52] is assessed additionally. IMWG defined stringent CR is not assessed and IMWG defined relapse from CR is not considered for PFS calculation. Instead, the IMWG definition of PD is also used for PFS calculation of patients with CR.

### Discontinuation criteria

#### Discontinuation of trial participation of individual patients

In the case of any of the following events, trial participation of the patient in question is discontinued: patient wishes to discontinue study participation; continued study participation is disadvantageous for the patient according to the investigator; emergence of an exclusion criterion that precludes further participation in the trial according to the principal investigator; emergence of a (serious) AE that precludes further participation in the trial; pregnancy (for female patients); incorrect data due to protocol violations by the patient (e.g. non-compliance); confirmed PD or PD causing end organ damage (CRAB criteria [53]), with the exception of asymptomatic PD after stem cell apheresis; trial completion according to the protocol.

#### Closure of individual trial sites

The principal investigator may prematurely close individual trial sites in the case of inadequate recruiting or data quality provided by the trial site in question.

#### Premature termination of the trial

The trial may be terminated prematurely by the principal investigator or the DSMB in the case of previously unknown risks or inadequate recruiting.

### Statistical analysis

#### Power calculation

The calculated number of total trial patients required to prove clinically relevant inferiority of PFS in arm A vs. arm B at a power of 80 % is 282. This power calculation is based on the formula of *Schoenfeld* [54] and the following assumptions: median PFS in arm A of 11 months (see prescribing information of lenalidomide); median PFS in arm B of 16.5 months (HR 0.67); Type 1 error of α = 0.05 with an α-spending according to *O’Brien* and *Fleming* [55] of 0.0052 for the planned interim analysis and 0.048 for the final analysis; power (1-β) of 0.8; 1:1 randomization; constant HR; interim analysis (O’Brien-Fleming Boundaries [55]); 15 % loss to follow up/non-compliance.

#### Study populations for analyses

Efficacy analyses are performed on an intent-to-treat (ITT) basis. The ITT population consists of all randomized patients. Patients with severe violation of inclusion/exclusion criteria are excluded. Patients are analyzed according to their randomization result. Safety analyses (toxicity, tolerability, medication) are performed on all patients that have received at least one administration of treatment according to the protocol. Patients are analyzed according to the received treatment.

#### Statistical methods

The primary objective (PFS) is analyzed on a confirmatory basis at a two-sided significance level of α = 0.048, which represents a significance level of α = 0.05 with adjustment for an interim analysis. A two-sided, stratified logrank test is applied with study site and first-line HDCT/ASCT (yes or no) as variables for stratification. An unstratified logrank test and a multivariate proportional hazard Cox regression model are calculated on an exploratory basis. Secondary objectives are analyzed on a descriptive or exploratory basis. For hypothesis-generating tests explicit p values are given without adjustment of the significance level for multiple testing and therefore reflect Type 1 errors related to the individual comparison and not the overall experiment. OS is analyzed analogously to PFS. Distribution of PFS and OS is estimated according to the method of Kaplan-Meier. Known prognostic factors for PFS and OS such as β2M and the number of prior treatments are analyzed in a multivariate Cox model. For time to best response cumulative incidence rates are calculated with disease progression and death as competing risks. Multivariate analyses including the comparison of time to best response in both trial arms are performed using the proportional hazards model for competing risks according to *Fine* and *Gray* [56]. The sole purpose of this analysis is the undistorted modeling of time to best response and not the estimation of competing risks. PFS and OS according to remission status before/after HDCT/ASCT are analyzed with landmark analyses (according to *Anderson* [57]) and Cox regression model including remission status (CR/VGPR: yes vs. no) as time-dependent co-variable. Treatment response rates are evaluated with Fisher’s exact test and Cochran/Armitage trend test. Demographic and clinical characteristics at enrollment are analyzed for homogeneity between both treatment groups. Toxicities in both treatment groups are compared in terms of type, frequency, CTC grading, and causality with Fisher’s exact test and Cochran/Armitage trend test.

#### Interim analysis

An interim analysis of preliminary efficacy (PFS) and safety (AEs) of the experimental trial arm (arm B) is performed after 96 PFS relevant events. The results are presented to the DSMB confidentially. The principal investigator only gains insight if the DSMB recommends to close the trial early or to alter the trial protocol. Additionally, the DSMB receives an annual report of the number of (serious) AEs and the number and severity of infections.

## Discussion

For RMM patients no universal therapeutic standard exists. Novel agents, especially lenalidomide in combination with dexamethasone are well evaluated [8, 9] and widely used in RMM patients. However, prospective data on salvage HDCT/ASCT are limited [49] and no prospective, randomized trial evaluating the benefit of salvage HDCT/ASCT versus continued novel agent-based salvage treatment has been published. In the ReLApsE trial, HDCT/ASCT is integrated into and compared against the widely used Rd salvage regimen. Due to the fact that patients randomized into the continued Rd arm are recommended to receive salvage HDCT/ASCT for their next relapse, comparison of early versus late salvage HDCT/ASCT will be possible. Additionally, cytogenetic bone marrow analysis by iFISH is integrated into the diagnostic workup at trial screening which will allow for evaluation of the prognostic relevance of recurrent cytogenetic aberrations in the context of salvage HDCT/ASCT. The ReLApsE trial is therefore expected to generate clinically relevant information to help guide decision-making in the RMM setting.

## Additional files

Additional file 1: SPIRIT schedule. (XLS 923 kb) Additional file 2: SPIRIT checklist. (DOC 121 kb)

## Abbreviations

AE: adverse event
ANC: absolute neutrophil count
ASCT: autologous stem cell transplantation
CR: complete response
CRAB: calcium elevation/renal failure/anemia/bone lesions
CT: computed tomography
CTCAE: Common Terminology Criteria of Adverse Events
DSMB: data safety monitoring board
EBMT: European Group for Bone and Marrow Transplantation
ECG: electrocardiogram
FPI: first patient in
HDCT: high dose chemotherapy
ICH-GCP: International Conference on Harmonization of good clinical practice
iFISH: interphase fluorescence in situ hybridization
IMiD: immunomodulatory drug
IMWG: International Myeloma Working Group
IRB: institutional review board
ITT: intent-to-treat
LMWH: low molecular weight heparin
LPI: last patient in
LPO: last patient out
MM: multiple myeloma
MR: minimal response
MRI: magnetic resonance imaging
ORR: overall response rate
OS: overall survival
PAD: bortezomib/doxorubicin/dexamethasone
PD: progressive disease
PFS: progression free survival
PI: proteasome inhibitor
PS: performance score
Rd: lenalidomide/dexamethasone
RMM: relapsed multiple myeloma
RR: response rate
SAE: serious adverse event
SUSAR: suspected unexpected serious adverse reaction
TRM: transplantation associated mortality
TTP: time to progression
VGPR: very good partial response
WHO: World health organization

## References

[1] Palumbo A, Anderson K (2011). Multiple myeloma. N Engl J Med.

[2] Cavo M, Rajkumar SV, Palumbo A, Moreau P, Orlowski R, Blade J, Sezer O, Ludwig H, Dimopoulos MA, Attal M, Sonneveld P, Boccadoro M, Anderson KC, Richardson PG, Bensinger W, Johnsen HE, Kroeger N, Gahrton G, Bergsagel PL, Vesole DH, Einsele H, Jagannath S, Niesvizky R, Durie BGM, San Miguel J, Lonial S (2011). International Myeloma Working Group consensus approach to the treatment of multiple myeloma patients who are candidates for autologous stem cell transplantation. Blood.

[3] Engelhardt M, Terpos E, Kleber M, Gay F, Wasch R, Morgan G, Cavo M, van de Donk N, Beilhack A, Bruno B, Johnsen HE, Hajek R, Driessen C, Ludwig H, Beksac M, Boccadoro M, Straka C, Brighen S, Gramatzki M, Larocca A, Lokhorst H, Magarotto V, Morabito F, Dimopoulos MA, Einsele H, Sonneveld P, Palumbo A (2014). European Myeloma Network recommendations on the evaluation and treatment of newly diagnosed patients with multiple myeloma. Haematologica.

[4] Dimopoulos MA, Richardson PG, Moreau P, Anderson KC (2014). Current treatment landscape for relapsed and/or refractory multiple myeloma. Nat Rev Clin Oncol.

[5] Singhal S, Mehta J, Desikan R, Ayers D, Roberson P, Eddlemon P, Munshi N, Anaissie E, Wilson C, Dhodapkar M, Zeddis J, Barlogie B (1999). Antitumor activity of thalidomide in refractory multiple myeloma. N Engl J Med.

[6] Palumbo A, Falco P, Ambrosini MT, Petrucci MT, Musto P, Caravita T, Pregno P, Bertola A, Cavallo F, Ciccone G, Boccadoro M (2005). Thalidomide plus dexamethasone is an effective salvage regimen for myeloma patients relapsing after autologous transplant. Eur J Haematol.

[7] Richardson PG, Sonneveld P, Schuster MW, Irwin D, Stadtmauer EA, Facon T, Harousseau J-L, Ben-Yehuda D, Lonial S, Goldschmidt H, Reece D, San-Miguel JF, Bladé J, Boccadoro M, Cavenagh J, Dalton WS, Boral AL, Esseltine DL, Porter JB, Schenkein D, Anderson KC (2005). Bortezomib or high-dose dexamethasone for relapsed multiple myeloma. N Engl J Med.

[8] Weber DM, Chen C, Niesvizky R, Wang M, Belch A, Stadtmauer EA, Siegel D, Borrello I, Rajkumar SV, Chanan-Khan AA, Lonial S, Yu Z, Patin J, Olesnyckyj M, Zeldis JB, Knight RD (2007). Lenalidomide plus dexamethasone for relapsed multiple myeloma in North America. N Engl J Med.

[9] Dimopoulos M, Spencer A, Attal M, Prince HM, Harousseau J-L, Dmoszynska A, San Miguel J, Hellmann A, Facon T, Foà R, Corso A, Masliak Z, Olesnyckyj M, Yu Z, Patin J, Zeldis JB, Knight RD (2007). Lenalidomide plus dexamethasone for relapsed or refractory multiple myeloma. N Engl J Med.

[10] San Miguel J, Weisel K, Moreau P, Lacy M, Song K, Delforge M, Karlin L, Goldschmidt H, Banos A, Oriol A, Alegre A, Chen C, Cavo M, Garderet L, Ivanova V, Martinez-Lopez J, Belch A, Palumbo A, Schey S, Sonneveld P, Yu X, Sternas L, Jacques C, Zaki M, Dimopoulos M (2013). Pomalidomide plus low-dose dexamethasone versus high-dose dexamethasone alone for patients with relapsed and refractory multiple myeloma (MM-003): a randomised, open-label, phase 3 trial. Lancet Oncol.

[11] Stewart AK, Rajkumar SV, Dimopoulos MA, Masszi T, Špička I, Oriol A, Hájek R, Rosiñol L, Siegel DS, Mihaylov GG, Goranova-Marinova V, Rajnics P, Suvorov A, Niesvizky R, Jakubowiak AJ, San-Miguel JF, Ludwig H, Wang M, Maisnar V, Minarik J, Bensinger WI, Mateos M-V, Ben-Yehuda D, Kukreti V, Zojwalla N, Tonda ME, Yang X, Xing B, Moreau P, Palumbo A (2014). Carfilzomib, Lenalidomide, and Dexamethasone for Relapsed Multiple Myeloma. N Engl J Med.

[12] Marriott JB, Dredge K, Dalgleish AG (2003). Thalidomide derived immunomodulatory drugs (IMiDs) as potential therapeutic agents. Curr Drug Targets Immune Endocr Metabol Disord.

[13] Kotla V, Goel S, Nischal S, Heuck C, Vivek K, Das B, Verma A (2009). Mechanism of action of lenalidomide in hematological malignancies. J Hematol Oncol.

[14] Quach H, Ritchie D, Stewart AK, Neeson P, Harrison S, Smyth MJ, Prince HM (2010). Mechanism of action of immunomodulatory drugs (IMiDS) in multiple myeloma. Leukemia.

[15] Hideshima T, Chauhan D, Podar K, Schlossman RL, Richardson P, Anderson KC (2001). Novel therapies targeting the myeloma cell and its bone marrow microenvironment. Semin Oncol.

[16] Dredge K, Marriott JB, Macdonald CD, Man H-W, Chen R, Muller GW, Stirling D, Dalgleish AG (2002). Novel thalidomide analogues display anti-angiogenic activity independently of immunomodulatory effects. Br J Cancer.

[17] Dredge K, Horsfall R, Robinson SP, Zhang L-H, Lu L, Tang Y, Shirley MA, Muller G, Schafer P, Stirling D, Dalgleish AG, Bartlett JB (2005). Orally administered lenalidomide (CC-5013) is anti-angiogenic in vivo and inhibits endothelial cell migration and Akt phosphorylation in vitro. Microvasc Res.

[18] Corral LG, Haslett PA, Muller GW, Chen R, Wong LM, Ocampo CJ, Patterson RT, Stirling DI, Kaplan G (1999). Differential cytokine modulation and T cell activation by two distinct classes of thalidomide analogues that are potent inhibitors of TNF-alpha. J Immunol.

[19] Davies FE, Raje N, Hideshima T, Lentzsch S, Young G, Tai YT, Lin B, Podar K, Gupta D, Chauhan D, Treon SP, Richardson PG, Schlossman RL, Morgan GJ, Muller GW, Stirling DI, Anderson KC (2001). Thalidomide and immunomodulatory derivatives augment natural killer cell cytotoxicity in multiple myeloma. Blood.

[20] Ito T, Ando H, Suzuki T, Ogura T, Hotta K, Imamura Y, Yamaguchi Y, Handa H (2010). Identification of a primary target of thalidomide teratogenicity. Science.

[21] Zhu YX, Braggio E, Shi C-X, Bruins LA, Schmidt JE, Van Wier S, Chang X-B, Bjorklund CC, Fonseca R, Bergsagel PL, Orlowski RZ, Stewart AK (2011). Cereblon expression is required for the antimyeloma activity of lenalidomide and pomalidomide. Blood.

[22] Heintel D, Rocci A, Ludwig H, Bolomsky A, Caltagirone S, Schreder M, Pfeifer S, Gisslinger H, Zojer N, Jäger U, Palumbo A (2013). High expression of cereblon (CRBN) is associated with improved clinical response in patients with multiple myeloma treated with lenalidomide and dexamethasone. Br J Haematol.

[23] Dimopoulos MA, Chen C, Spencer A, Niesvizky R, Attal M, Stadtmauer EA, Petrucci MT, Yu Z, Olesnyckyj M, Zeldis JB, Knight RD, Weber DM (2009). Long-term follow-up on overall survival from the MM-009 and MM-010 phase III trials of lenalidomide plus dexamethasone in patients with relapsed or refractory multiple myeloma. Leukemia.

[24] Rajkumar SV, Jacobus S, Callander NS, Fonseca R, Vesole DH, Williams ME, Abonour R, Siegel DS, Katz M, Greipp PR (2010). Lenalidomide plus high-dose dexamethasone versus lenalidomide plus low-dose dexamethasone as initial therapy for newly diagnosed multiple myeloma: an open-label randomised controlled trial. Lancet Oncol.

[25] Miguel J, Dimopoulos M, Weber D, Olesnyckyi M, Yu Z, Zeldis J (2007). Dexamethasone dose adjustmenst seem to result in better efficacy and improved tolerability in patients with relapsed/refractory multiple myeloma who are treated with lenalidomide/dexamethasone (MM009/010 Sub-Analysis). ASH Annu Meet Abstr.

[26] Attal M, Lauwers-Cances V, Marit G, Caillot D, Moreau P, Facon T, Stoppa AM, Hulin C, Benboubker L, Garderet L, Decaux O, Leyvraz S, Vekemans M-C, Voillat L, Michallet M, Pegourie B, Dumontet C, Roussel M, Leleu X, Mathiot C, Payen C, Avet-Loiseau H, Harousseau J-L (2012). Lenalidomide maintenance after stem-cell transplantation for multiple myeloma. N Engl J Med.

[27] McCarthy PL, Owzar K, Hofmeister CC, Hurd DD, Hassoun H, Richardson PG, Giralt S, Stadtmauer EA, Weisdorf DJ, Vij R, Moreb JS, Callander NS, Van Besien K, Gentile T, Isola L, Maziarz RT, Gabriel DA, Bashey A, Landau H, Martin T, Qazilbash MH, Levitan D, McClune B, Schlossman R, Hars V, Postiglione J, Jiang C, Bennett E, Barry S, Bressler L (2012). Lenalidomide after stem-cell transplantation for multiple myeloma. N Engl J Med.

[28] Palumbo A, Cavallo F, Gay F, Di Raimondo F, Ben Yehuda D, Petrucci MT, Pezzatti S, Caravita T, Cerrato C, Ribakovsky E, Genuardi M, Cafro A, Marcatti M, Catalano L, Offidani M, Carella AM, Zamagni E, Patriarca F, Musto P, Evangelista A, Ciccone G, Omedé P, Crippa C, Corradini P, Nagler A, Boccadoro M, Cavo M (2014). Autologous Transplantation and Maintenance Therapy in Multiple Myeloma. N Engl J Med.

[29] Palumbo A, Hajek R, Delforge M, Kropff M, Petrucci MT, Catalano J, Gisslinger H, Wiktor-Jędrzejczak W, Zodelava M, Weisel K, Cascavilla N, Iosava G, Cavo M, Kloczko J, Bladé J, Beksac M, Spicka I, Plesner T, Radke J, Langer C, Ben Yehuda D, Corso A, Herbein L, Yu Z, Mei J, Jacques C, Dimopoulos MA (2012). Continuous lenalidomide treatment for newly diagnosed multiple myeloma. N Engl J Med.

[30] Palumbo A, Bringhen S, Kumar SK, Lupparelli G, Usmani S, Waage A, Larocca A, van der Holt B, Musto P, Offidani M, Petrucci MT, Evangelista A, Zweegman S, Nooka AK, Spencer A, Dimopoulos MA, Hajek R, Cavo M, Richardson P, Lonial S, Ciccone G, Boccadoro M, Anderson K, Barlogie B, Sonneveld P, McCarthy PL (2014). Second primary malignancies with lenalidomide therapy for newly diagnosed myeloma: a meta-analysis of individual patient data. Lancet Oncol.

[31] Ludwig H, Miguel JS, Dimopoulos MA, Palumbo A, Garcia Sanz R, Powles R, Lentzsch S, Ming Chen W, Hou J, Jurczyszyn A, Romeril K, Hajek R, Terpos E, Shimizu K, Joshua D, Hungria V, Rodriguez Morales A, Ben-Yehuda D, Sondergeld P, Zamagni E, Durie B (2014). International Myeloma Working Group recommendations for global myeloma care. Leukemia.

[32] Tricot G, Jagannath S, Vesole DH, Crowley J, Barlogie B (1995). Relapse of multiple myeloma after autologous transplantation: survival after salvage therapy. Bone Marrow Transplant.

[33] Vesole DH, Crowley JJ, Catchatourian R, Stiff PJ, Johnson DB, Cromer J, Salmon SE, Barlogie B (1999). High-dose melphalan with autotransplantation for refractory multiple myeloma: results of a Southwest Oncology Group phase II trial. J Clin Oncol.

[34] Rajkumar SV, Fonseca R, Lacy MQ, Witzig TE, Lust JA, Greipp PR, Therneau TM, Kyle RA, Litzow MR, Gertz MA (1999). Autologous stem cell transplantation for relapsed and primary refractory myeloma. Bone Marrow Transplant.

[35] Lee C-K, Barlogie B, Zangari M, Fassas A, Anaissie E, Morris C, Van Rhee F, Cottler-Fox M, Thertulien R, Muwalla F, Mazher S, Badros A, Tricot G (2002). Transplantation as salvage therapy for high-risk patients with myeloma in relapse. Bone Marrow Transplant.

[36] Alvares CL, Davies FE, Horton C, Patel G, Powles R, Morgan GJ (2006). The role of second autografts in the management of myeloma at first relapse. Haematologica.

[37] Fenk R, Liese V, Neubauer F, Bruns I, Kondakci M, Balleisen S, Saure C, Schröder T, Haas R, Kobbe G (2011). Predictive factors for successful salvage high-dose therapy in patients with multiple myeloma relapsing after autologous blood stem cell transplantation. Leuk Lymphoma.

[38] Cook G, Liakopoulou E, Pearce R, Cavet J, Morgan GJ, Kirkland K, Lee J, Davies FE, Hall R, Rahemtulla A, Russell N, Marks DI (2011). Factors influencing the outcome of a second autologous stem cell transplant (ASCT) in relapsed multiple myeloma: a study from the British Society of Blood and Marrow Transplantation Registry. Biol Blood Marrow Transplant.

[39] Jimenez-Zepeda VH, Mikhael J, Winter A, Franke N, Masih-Khan E, Trudel S, Chen C, Kukreti V, Reece DE (2012). Second autologous stem cell transplantation as salvage therapy for multiple myeloma: impact on progression-free and overall survival. Biol Blood Marrow Transplant.

[40] Lemieux E, Hulin C, Caillot D, Tardy S, Dorvaux V, Michel J, Gastinne T, Rossi C, Legouge C, Touzeau C, Planche L, Loirat M, Lafon I, Moreau P (2013). Autologous stem cell transplantation: an effective salvage therapy in multiple myeloma. Biol Blood Marrow Transplant.

[41] Sellner L, Heiss C, Benner A, Raab MS, Hillengass J, Hose D, Lehners N, Egerer G, Ho AD, Goldschmidt H, Neben K (2013). Autologous retransplantation for patients with recurrent multiple myeloma: a single-center experience with 200 patients. Cancer.

[42] Gonsalves WI, Gertz MA, Lacy MQ, Dispenzieri A, Hayman SR, Buadi FK, Dingli D, Hogan WJ, Kumar SK (2013). Second auto-SCT for treatment of relapsed multiple myeloma. Bone Marrow Transplant.

[43] Michaelis LC, Saad A, Zhong X, Le-Rademacher J, Freytes CO, Marks DI, Lazarus HM, Bird JM, Holmberg L, Kamble RT, Kumar S, Lill M, Meehan KR, Saber W, Schriber J, Tay J, Vogl DT, Wirk B, Savani BN, Gale RP, Vesole DH, Schiller GJ, Abidi M, Anderson KC, Nishihori T, Kalaycio ME, Vose JM, Moreb JS, Drobyski W, Munker R (2013). Salvage second hematopoietic cell transplantation in myeloma. Biol Blood Marrow Transplant.

[44] Auner HW, Szydlo R, Rone A, Chaidos A, Giles C, Kanfer E, Macdonald DH, Marin D, Milojkovic D, Pavlu J, Apperley JF, Rahemtulla A (2013). Salvage autologous stem cell transplantation for multiple myeloma relapsing or progressing after up-front autologous transplantation. Leuk Lymphoma.

[45] Singh Abbi KK, Zheng J, Devlin SM, Giralt S, Landau H (2015). Second autologous stem cell transplant: an effective therapy for relapsed multiple myeloma. Biol Blood Marrow Transplant.

[46] Fermand JP, Ravaud P, Chevret S, Divine M, Leblond V, Belanger C, Macro M, Pertuiset E, Dreyfus F, Mariette X, Boccacio C, Brouet JC (1998). High-dose therapy and autologous peripheral blood stem cell transplantation in multiple myeloma: up-front or rescue treatment? Results of a multicenter sequential randomized clinical trial. Blood.

[47] Barlogie B, Kyle RA, Anderson KC, Greipp PR, Lazarus HM, Hurd DD, McCoy J, Moore DF, Dakhil SR, Lanier KS, Chapman RA, Cromer JN, Salmon SE, Durie B, Crowley JC (2006). Standard chemotherapy compared with high-dose chemoradiotherapy for multiple myeloma: final results of phase III US Intergroup Trial S9321. J Clin Oncol.

[48] Atanackovic D, Schilling G (2013). Second autologous transplant as salvage therapy in multiple myeloma. Br J Haematol.

[49] Cook G, Williams C, Brown JM, Cairns DA, Cavenagh J, Snowden JA, Ashcroft AJ, Fletcher M, Parrish C, Yong K, Cavet J, Hunter H, Bird JM, Chalmers A, O’Connor S, Drayson MT, Morris TCM (2014). High-dose chemotherapy plus autologous stem-cell transplantation as consolidation therapy in patients with relapsed multiple myeloma after previous autologous stem-cell transplantation (NCRI Myeloma X Relapse [Intensive trial]): a randomised, open-label. Lancet Oncol.

[50] Neben K, Jauch A, Bertsch U, Heiss C, Hielscher T, Seckinger A, Mors T, Müller NZ, Hillengass J, Raab MS, Ho AD, Hose D, Goldschmidt H (2010). Combining information regarding chromosomal aberrations t(4;14) and del(17p13) with the International Staging System classification allows stratification of myeloma patients undergoing autologous stem cell transplantation. Haematologica.

[51] Durie BGM, Harousseau J-L, Miguel JS, Bladé J, Barlogie B, Anderson K, Gertz M, Dimopoulos M, Westin J, Sonneveld P, Ludwig H, Gahrton G, Beksac M, Crowley J, Belch A, Boccadaro M, Cavo M, Turesson I, Joshua D, Vesole D, Kyle R, Alexanian R, Tricot G, Attal M, Merlini G, Powles R, Richardson P, Shimizu K, Tosi P, Morgan G (2006). International uniform response criteria for multiple myeloma. Leukemia.

[52] Bladé J, Samson D, Reece D, Apperley J, Björkstrand B, Gahrton G, Gertz M, Giralt S, Jagannath S, Vesole D (1998). Criteria for evaluating disease response and progression in patients with multiple myeloma treated by high-dose therapy and haemopoietic stem cell transplantation. Myeloma Subcommittee of the EBMT. European Group for Blood and Marrow Transplant. Br J Haematol.

[53] The International Myeloma Working Group (2003). Criteria for the classification of monoclonal gammopathies, multiple myeloma and related disorders: a report of the International Myeloma Working Group. Br J Haematol.

[54] Schoenfeld D (1981). The asymptotic properties of nonparametric tests for comparing survival distributions. Biometrika.

[55] O’Brien PC, Fleming TR (1979). A multiple testing procedure for clinical trials. Biometrics.

[56] Fine JP, Gray RJ (1999). A proportional hazards model for the subdistribution of a competing risk. J Am Stat Assoc.

[57] Anderson JR, Cain KC, Gelber RD (1983). Analysis of survival by tumor response. J Clin Oncol.

[58] Durie BG, Salmon SE (1975). A clinical staging system for multiple myeloma. Correlation of measured myeloma cell mass with presenting clinical features, response to treatment, and survival. Cancer.

